# Optical Coherence Tomography for Assessing the Severity of Dental Caries: An In Vitro Validation Study

**DOI:** 10.3390/dj13110543

**Published:** 2025-11-20

**Authors:** In-Kyung Hwang, Sun-Young Kim, Tae-Il Kim

**Affiliations:** 1 Department of Periodontology, Research Institute for Dental Engineering, Gangneung-Wonju National University College of Dentistry, Gangneung 25457, Republic of Korea; perioikh@gwnu.ac.kr; 2 Department of Conservative Dentistry, Dental Research Institute, Seoul National University School of Dentistry, Seoul 03080, Republic of Korea; denkim@snu.ac.kr; 3 Department of Periodontology, Dental Research Institute, Seoul National University School of Dentistry, Seoul 03080, Republic of Korea

**Keywords:** dental caries, optical coherence tomography, oral diagnosis, tooth demineralization

## Abstract

**Background/Objectives**: Differentiating initial from moderate non-cavitated occlusal caries using intraoral radiography is challenging. This in vitro study aimed to verify the ability of Optical Coherence Tomography (OCT) to discern the extent of demineralization in non-cavitated carious lesions and discriminate between the exact caries stages. **Methods**: In total, 110 extracted molars and premolars with occlusal caries were examined by two calibrated examiners using OCT and radiography. Histological sections stained with acid red were used as the reference standard. Diagnostic accuracy was calculated by comparing OCT- and radiograph-based diagnoses with the histologic reference standard. **Results:** OCT demonstrated superior sensitivity (0.83), specificity (0.76), and overall diagnostic accuracy (0.79) for distinguishing moderate from initial lesions, outperforming intraoral radiography, which achieved a sensitivity of 0.48, specificity of 0.84, and accuracy of 0.70. McNemar’s test showed a significant difference in sensitivity (*p* < 0.05), but not in specificity (*p* > 0.05), between the two diagnostic methods. **Conclusions:** These findings confirm that OCT can visualize caries progression with sufficient precision to distinguish between the initial and moderate lesion stages in an in vitro setting. Further validation in clinical trials is necessary to support OCT’s application for routine caries diagnosis.

## 1. Introduction

Accurate and early detection of dental caries remains a significant challenge in the field of conservative dentistry, particularly when distinguishing between the various stages of caries development. Dental caries, a prevalent chronic disease affecting a large portion of the global population, can progress from initial and moderate non-cavitated lesions to more severe cavitated stages if not appropriately diagnosed and managed [1]. Although several diagnostic tools such as fluorescence imaging or transillumination have been developed, caries detection relies heavily on visual–tactile examination and radiographic methods, which, while effective, have notable limitations in sensitivity, particularly for detecting non-severe caries [2]. A recent consensus report from the European Organization for Caries Research and the European Federation of Conservative Dentistry (ORCA-EFCD) concluded that visual examination remains the first choice for caries detection, with intraoral radiography recommended as the primary diagnostic tool [3]. Intraoral radiography is the most commonly used diagnostic method; however, its performance varies depending on the location and stage of the lesion. It is beneficial for detecting minimally cavitated proximal caries compared to visual examination, but less sensitive for detecting non-cavitated occlusal caries [4]. Even when non-cavitated lesions are detected by visual examination, clinicians often face the challenge of determining whether they require treatment because their ambiguous nature complicates clinical decision-making.

Several scoring systems for dental caries have been developed to enhance objectivity in diagnosis, ensure consistency, and standardize treatment recommendations [5,6,7,8]. Among these, the International Caries Detection and Assessment System (ICDAS) has become the most widely used classification system, providing a structured framework for assessing caries severity through visual examination [6,9]. In 2019, advances in caries diagnosis led to the introduction of CariesCare International (CCI), an evolved framework from the ICDAS Foundation designed to improve clinical applicability and patient-centered care in the original ICDAS [10]. Based on the ICDAS, which categorizes lesions on a detailed 1-to-6 scale, CCI simplifies the classification by focusing on three primary stages of lesion progression: initial, moderate, and extensive, corresponding to ICDAS codes 1–2, 3–4, and 5–6, respectively.

For extensive carious lesions, obvious dentin cavitations are easily detected through visual examination, and noticeable radiolucency on radiographs readily reveals the lesions, making surgical intervention evident. In contrast, initial and moderate non-cavitated lesions pose diagnostic challenges, as clinicians often face uncertainties regarding lesion depth and caries activity. This ambiguity complicates decisions on whether to employ minimally invasive surgical treatment to prevent progression or whether periodic monitoring is sufficient. Given that caries diagnosis traditionally relies on conventional methods such as visual–tactile inspection and radiography, there is a pressing need for more advanced diagnostic tools that can reliably differentiate between initial and moderate carious lesions [11].

Optical Coherence Tomography (OCT) has emerged as a promising technology, offering a noninvasive imaging method that provides high-resolution cross-sectional images of dental tissues. Originally developed as a diagnostic tool in ophthalmology, OCT uses the principles of low-coherence interferometry, where light waves are directed into the tissue and reflections from different depths are analyzed to create detailed cross-sectional images [12]. This technique allows for the precise visualization of internal tissue structures. In dentistry, OCT can detect structural defects in teeth and identify periodontal bone loss [13,14,15,16]. Although previous studies have demonstrated the ability of OCT to detect caries, research specifically focused on the use of OCT to differentiate the stages of caries progression remains limited [14,17,18].

This study explored the potential of OCT for diagnosing dental caries by visualizing subsurface lesions that are often undetectable using conventional methods. We hypothesized that OCT will achieve greater diagnostic accuracy than intraoral radiographs for staging non-cavitated occlusal caries by directly visualizing the extent of demineralization. Therefore, this study aimed to validate a novel OCT-based diagnostic device for discriminating between initial and moderate carious lesions, and to compare its accuracy with conventional intraoral radiography.

## 2. Materials and Methods

This study was designed to compare the diagnostic accuracy of two approaches for detecting and assessing carious lesions: (1) visual inspection combined with OCT, and (2) visual inspection combined with intraoral radiography. One hundred and ten extracted molar and premolar teeth were selected for analysis, presenting with suspected non-cavitated carious lesions. Two calibrated examiners (I.K.H. and S.Y.K.) independently examined each tooth using both diagnostic methods. The initial and moderate stage caries were defined as shown in Table 1, according to the CCI standard [9], and correlated the stages of caries with the actual lesion depth from previous studies [19,20,21]. Based on these criteria, the caries stage of each tooth was determined during the experiment.

The primary objective of this study was to determine whether carious lesions could be accurately classified as initial or moderate lesions based on the results of the two diagnostic approaches. As a secondary objective, this study aimed to assess the inter-rater reliability of both methods to evaluate the consistency between the two examiners’ diagnoses.

To establish a reliable gold standard, histological verification was performed on each tooth following diagnostic assessments. Based on the confirmed lesion depth and condition, the true severity of each lesion was determined as either initial or moderate. The diagnostic outcomes from the OCT and radiographic assessments were then compared with the histologically verified outcomes to evaluate the accuracy of each method.

### 2.1. Sample Preparation

Teeth were obtained from the Biobank of Seoul National University Dental Hospital and Seoul Dental Hospital for the Disabled, following approval of the study protocol by the Institutional Review Board of Seoul National University Dental Hospital (IRB No. CRI123015; approval date: 4 December 2023). All samples were collected with informed consent from the patients, following the ethical guidelines for research on human tissues, including the completion of the anonymization process. The teeth were stored in a 4% Chloramine T solution and cleaned with saline before being prepared as specimens. Each tooth was embedded in a 2 × 2 × 2 cm^3^ acryl mold using a hydrophilic vinyl polysiloxane impression material (EXAFINE PUTTY TYPE^®^, GC Corporation, Tokyo, Japan) for further in vitro analysis.

A dentist with 3 years of experience in conservative dentistry (S.M.K.) selected the teeth. The dentist inspected and selected teeth exhibiting non-cavitated lesions, with ICDAS scores ranging from 1 to 4. We focused exclusively on non-cavitated lesions because differentiating between initial lesions requiring only preventive care and moderate lesions needing restorative treatment remains a diagnostic challenge with conventional methods. Lesions were confined to occlusal caries in molars and premolars to reduce anatomical variability and standardize OCT image interpretation, consistent with previous OCT validation studies [17,22]. Teeth with visible carious cavities (ICDAS 5–6), existing restorations, significant fractures, hypoplasia, or other developmental defects were excluded from the study to maintain consistency in lesion type.

The sample size was determined using a formula for diagnostic accuracy studies, considering the expected sensitivity and specificity of the diagnostic methods. Based on a systematic review of emerging technologies for detecting non-cavitated dental caries, an expected sensitivity of 0.8, 95% confidence interval, prevalence of 60%, and 10% margin of error were assumed for the calculation [23]. Using Buderer’s formula [24], a minimum of 103 examination sites were required, and 110 examination sites were included in the study to account for potential sample loss.

### 2.2. OCT with Visual Inspection

**Figure 1:**
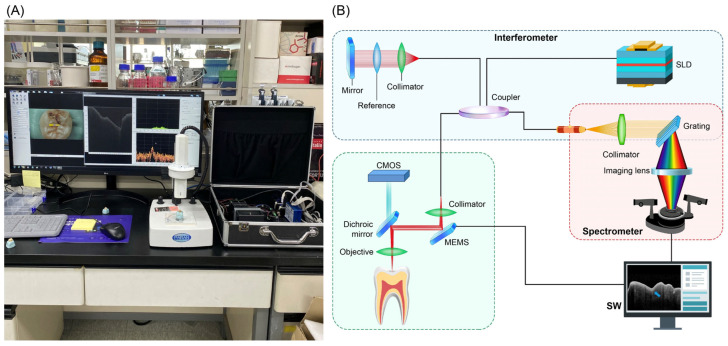
(**A**) Benchtop spectral-domain optical coherence tomography instrument used in this study. (**B**) Schematic of image formation (CMOS, complementary metal–oxide–semiconductor; MEMS, microelectromechanical system; SLD, superluminescent diode; SW, software).

The OCT system (WINUS Technology, Gunpo, Republic of Korea) used in this study was a benchtop prototype for in vitro experiments with a planned pen-type clinical version (Figure 1A). Broadband light from a superluminescent diode (SLD; center wavelength of 840 nm) was split into reference and sample arms by a fiber coupler. In the reference arm, the beam was collimated and reflected using a mirror. In the sample arm, a collimator, microelectromechanical system (MEMS) scanner, and objective lens delivered the beam to the tooth and collected the backscattered light. A dichroic mirror directed visible light to a coaxial complementary metal–oxide–semiconductor (CMOS) camera for surface guidance. The combined interferometric signal was sent to a spectrometer, where the collimator, grating, and imaging lens were dispersed in the spectrum for detection (Figure 1B). Spectral data were processed using a Fourier transform to reconstruct depth-resolved A-scans and cross-sectional B-scans. The A-scan rate was 50 kHz, and the axial and lateral resolutions were 10 µm and 20 µm, respectively.

Before the main diagnostic assessments, the two examiners (I.K.H. and S.Y.K.) were calibrated for OCT interpretation using 10 separately prepared teeth: 5 with initial-stage lesions and 5 with moderate-stage lesions. For each specimen, they reviewed the OCT B-scans and assigned a stage based on the extent of the hyperreflective lesion body, which appeared as a bright zone, and surface integrity. The lesion depth was verified by stepwise exposure using a high-speed round bur.

On OCT, demineralization appears as a localized hyperreflective zone accompanied by subsurface signal attenuation and shadowing. Surface integrity was assessed by the continuity of the surface line: a continuous line indicates an intact surface, while disruption indicates microcavitation. Lesion severity was classified based on the following quantitative parameters:

Initial lesions were defined by (1) hyperreflective bands limited to the outer/middle enamel layer and (2) an intact dentinoenamel junction (DEJ) with no signal penetration into dentin.

Moderate lesions were characterized by (1) hyperreflective bands extending beyond the DEJ into dentin and (2) presence of signal shadowing beneath the hyperreflective zone in the underlying dentin.

The DEJ served as the primary anatomical landmark for objective differentiation between lesion stages, with lesion depth relative to the DEJ and the presence of subsurface shadowing serving as quantifiable criteria [13].

After calibration, each of the 110 teeth was evaluated using a combination of visual inspection and OCT imaging. For visual inspection, each tooth was air-dried for 5 s to enhance the visibility of the carious lesions and examined under standardized lighting conditions. A dental mirror and ball-ended probe were used to detect surface changes associated with caries. After visual inspection, OCT images were obtained by scanning the occlusal surface along the buccolingual axis. The OCT system used in this study projects a swept laser onto the occlusal surface of each tooth. It provides cross-sectional images based on the light reflected from variations in the internal structure. The two examiners, I.K.H. and S.Y.K., graded each of the 110 teeth using the images obtained according to the written criteria in Table 1.

### 2.3. Intraoral Radiographs with Visual Inspection

Three months after the initial OCT examination, the two examiners (I.K.H. and S.Y.K.) conducted a second in vitro diagnosis using intraoral radiographs and visual inspection. This 3-month washout period was implemented to minimize potential operator bias and recall effects when evaluating the same 110 extracted teeth. As in the OCT-based assessment, each tooth was first visually inspected under standardized lighting conditions and evaluated using digital radiographic images obtained with an intraoral X-ray system (KaVo^TM^ Focus^TM^, KaVo Dental, Bieberich, Germany). Radiographs were obtained in the buccolingual direction under standardized settings of 60 kV and 7 mA, with an exposure time of 0.08 s. The examiners graded the caries status of each tooth using the same diagnostic criteria used in the previous examination.

### 2.4. Reference Test—Validation of the Actual Lesion Depth

After imaging, 110 extracted teeth were sectioned and examined histologically; this is the accepted reference standard for validating lesion depth in in vitro caries accuracy studies [25]. Diagnoses based on OCT and radiography were compared with histological references. Each tooth was cut longitudinally through the center of the identified lesion using a water-cooled diamond saw, ensuring that the integrity of the tissue structure was maintained. The cut surfaces were polished to facilitate precise observation of the lesion boundary. To eliminate potential bias, histological assessment was performed by an independent examiner (S.W.L.) who was blinded to the OCT and radiographic results. Specimens were coded numerically, and diagnoses based on OCT and radiography were compared with histological references only after all assessments were completed.

The sectioned teeth were treated with 0.5% acid red solution (Caries Detector, Kuraray, Medical Inc., Tokyo, Japan) for 10 s to visualize the carious lesions. The stained sections were examined under an endodontic microscope (Zeiss Extaro 300, Carl Zeiss Meditec AG, Oberkochen, Germany) to inspect the undecalcified tooth specimens at various magnifications ranging from 3× to 30×. The lesions were inspected to confirm the presence, extent, and depth of demineralization. Observations included measuring the thickness of the affected enamel and the extent of penetration into the dentin, allowing differentiation between initial and moderate lesions. Lesions were then categorized based on the depth criteria; demineralization confined to the enamel or less than the outer third of the dentin was classified as the initial stage, while demineralization penetrating more than the outer third of the dentin was classified as a moderate stage. Histological assessment served as the reference standard against which the diagnostic outcomes of OCT and intraoral radiography were compared.

### 2.5. Statistical Analysis

The diagnostic performances of the two approaches, visual inspection combined with OCT and visual inspection combined with intraoral radiography, were evaluated by comparing the assessments of the more experienced examiner, S.Y.K., against the histologically confirmed gold standard. The sensitivity, specificity, and overall accuracy were calculated for each method based on the S.Y.K. evaluations. McNemar’s test was used to compare the sensitivity and specificity of the two diagnostic approaches for the same set of teeth. Statistical significance was considered at a *p*-value of less than 0.05, indicating a meaningful difference in diagnostic accuracy between the methods. Inter-rater reliability was assessed using Cohen’s kappa coefficient (κ) to measure the level of agreement between the two examiners (S.Y.K. and I.K.H.) for each diagnostic method [26]. The degree of agreement was classified based on the following criteria: 0.81–1.00, almost perfect agreement; 0.61–0.80, substantial agreement; 0.41–0.60, moderate agreement; 0.21–0.40, fair agreement; 0.00–0.20, slight agreement. Statistical analyses were performed using IBM SPSS Statistics (version 26.0; IBM Corp., Armonk, NY, USA).

## 3. Results

**Figure 2:**
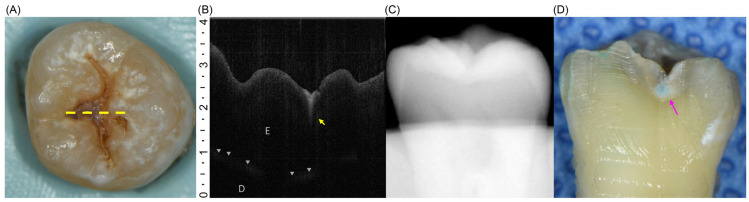
(**A**) Occlusal view of a tooth with an initial lesion. The yellow dotted line indicates the scan plane for OCT imaging. (**B**) OCT B-scan showing a slight surface irregularity and a hyperreflective zone (yellow arrow) confined to the enamel, not reaching the DEJ (gray triangles). D, dentin; E, enamel. (**C**) Intraoral radiograph showing no detectable radiolucency. (**D**) Sectioned specimens stained with 0.5% acid red solution. The pink arrow indicates demineralized area limited to the outer third of the dentin.

**Figure 3:**
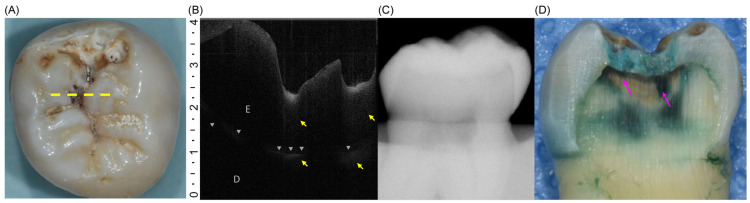
(**A**) Occlusal view of a tooth with a moderate lesion. The yellow dotted line indicates the scan plane for OCT imaging. (**B**) OCT B-scan showing a surface irregularity and a broad hyperreflective zone (yellow arrows) extending beyond the DEJ (gray triangles) with underlying shadowing in the dentin layer. D, dentin; E, enamel. (**C**) Intraoral radiograph showing no detectable radiolucency. (**D**) Sectioned specimens stained with 0.5% acid red solution. The pink arrow indicates demineralized area extending beyond the outer third of the dentin.

All 110 extracted teeth were assessed using both diagnostic approaches and classified as having initial or moderate lesions (Figure 2A and Figure 3A). However, during the histological reference test, three specimens were lost because of unintended fractures that occurred during sectioning. Consequently, the final analysis included 107 teeth, with the distribution of lesions confirmed by histology, showing 64 teeth with initial lesions and 43 teeth with moderate lesions (Table 2) (Figure 2D and Figure 3D). On OCT, the initial lesions appeared as localized hyperreflective bands confined to the enamel, with an intact DEJ and a continuous surface line (Figure 2B). Moderate lesions showed a broader hyperreflective zone crossing the DEJ with underlying shadowing and surface disruption (Figure 3B). In contrast, intraoral radiographs rarely displayed apparent radiolucency of the initial lesions and frequently showed no discernible change, even for moderate occlusal involvement (Figure 2C and Figure 3C).

**Figure 4:**
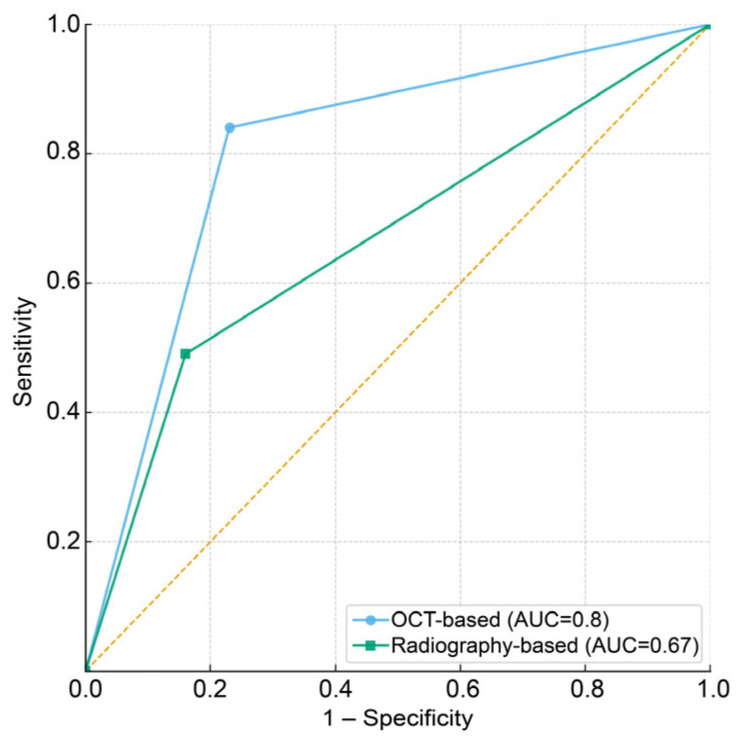
ROC curves comparing the diagnostic performance of OCT-based (blue line) and radiography-based (green line) methods. The AUC was 0.80 for the OCT method and 0.67 for the radiography method, demonstrating superior diagnostic discrimination by OCT (ROC, receiver operating characteristic; AUC, area under the curve).

The diagnostic performances of each method were compared in terms of sensitivity, specificity, overall accuracy, and area under the curve (AUC) values (Table 3). The AUC values and receiver operating characteristic (ROC) curves demonstrated that the OCT method achieved superior discrimination of caries severity compared to the radiographic method (Figure 4). The contingency table for overall accuracy indicates that the OCT-based method resulted in fewer incorrect diagnoses and more accurate diagnoses than the radiograph-based method (Table 4). Visual inspection combined with OCT showed an overall diagnostic accuracy of 0.79, with a sensitivity of 0.83 and specificity of 0.76. In contrast, the visual inspection combined with the intraoral radiographs approach exhibited an overall accuracy of 0.70, with a sensitivity of 0.48 and specificity of 0.84. A statistically significant difference in sensitivity was observed between the two methods by McNemar’s test (*p* = 0.002), indicating that OCT-based assessment was more effective in detecting moderate lesions. By contrast, the difference in specificity was not significant (*p* = 0.120), suggesting that both methods performed similarly in identifying initial lesions.

Inter-examiner reliability was evaluated with Cohen’s κ. Both methods showed substantial agreement: κ = 0.77 (95% CI 0.68–0.85) for visual inspection with OCT and κ = 0.81 (95% CI 0.73–0.88) for visual inspection with intraoral radiographs.

## 4. Discussion

This study’s results demonstrated the potential use of OCT as an advanced imaging modality capable of precisely visualizing subsurface carious lesions. Visual inspection combined with OCT demonstrated a higher overall diagnostic accuracy and sensitivity in distinguishing moderate from initial lesions than intraoral radiography. Previous studies have shown that OCT produces cross-sectional images that differentiate demineralized from sound tissues by increasing light scattering and reflectivity in porous demineralized regions [13,14,27]. This enhanced scattering appears as a bright zone, facilitating separation from sound tissue, whereas on intraoral radiographs, demineralization appears as radiolucency [17]. In this study, the caries severity was determined based on the extent of the hyperreflective region and surface integrity.

Advancing from previous caries detection research, this present study focused on distinguishing lesion stages because identifying moderate caries from initial lesions is essential for interventions that can halt caries progression with minimal operative treatment. OCT’s high sensitivity for detecting subtle differences in demineralization and structural changes suggests that it may be an effective tool for discerning lesion stages that are difficult to determine using conventional radiography. Prior OCT studies by Luong et al. and Shimada et al. staged non-severe lesions into three categories using three-dimensional reconstruction [17,28]. In contrast, this study adopted binary staging of non-severe lesions—initial and moderate—which aligns with the CariesCare practice guide by the CCI and updated diagnosis and treatment guidelines from the ICDAS [10]. CCI has also merged ICDAS codes 1–2 and 3–4 into the categories of initial and moderate lesions, respectively. Furthermore, this study specified the interpretation criteria for OCT images, the extent of the hyperreflective zone, and the integrity of the surface line. Although Luong and Shimada used 3D OCT images, which may add complexity without improving the clarity in some cases, the emphasis on easily interpreted B-scans supports the clinical applicability of OCT.

Several in vivo studies have validated OCT as a precise diagnostic tool for early caries detection, providing important context for our findings [29,30]. Clinical studies also demonstrate that OCT can serve as an active surveillance tool for dental caries, capable of monitoring remineralization in active lesions [31]. In clinical settings, localized enamel breakdown and dentin demineralization appear as bright zones in OCT images, consistent with our in vitro observations [22,29,30,31]. Notably, in vivo studies report OCT penetration depths of approximately 2.5 mm below the surface, enabling visualization of lesion depth relative to the DEJ—findings that align with our observation of demineralization patterns extending to the DEJ [29]. While in vivo studies provide valuable real-world diagnostic performance data, they typically cannot employ histological validation as the gold standard, since patients with non-cavitated caries receive preventive rather than operative treatment [22,29]. Our in vitro approach thus complements existing clinical studies by providing histologic validation that supports the diagnostic capabilities observed in clinical practice.

In this study, both OCT- and radiographic-based approaches showed substantial agreement of inter-rater reliability, though Cohen’s κ value was slightly lower for OCT-based analysis. These findings indicated a learning curve for OCT image interpretation, underscoring the need for thorough examiner training and calibration. Developing software to aid the interpretation of OCT images by classifying lesion severity and extent could improve the clinical usability of OCT. Furthermore, in vivo studies using more advanced, clinically adapted devices would help confirm the diagnostic utility of OCT.

While OCT demonstrated superior sensitivity compared to radiography, it exhibited slightly lower specificity, though this difference was not statistically significant. This marginally higher false-positive rate suggests that OCT may classify initial lesions as moderate lesions. Therefore, clinicians using OCT should interpret borderline findings conservatively, prioritizing preventive interventions and monitoring over immediate restorative treatment, particularly when clinical signs suggest early-stage disease. This approach aligns with minimally invasive dentistry principles while leveraging OCT’s superior detection capabilities.

Beyond caries detection, OCT is also valuable for identifying structural defects in dental restorations, particularly at the tooth–composite interface [13,32]. OCT can reveal interfacial defects because of its high-resolution imaging, which detects discrepancies in light reflection and scattering patterns at the boundaries of different materials. These imaging properties enable OCT to highlight subtle defects, such as air bubbles, microgaps, or adhesive failures, which may compromise the integrity of restorations. Similarly, OCT can be used to visualize cracks within the enamel and dentin layers without invasive preparation. When a crack is present, OCT detects an interruption in the continuity of the dental structure, which appears as a bright, well-defined line or gap in cross-sectional images [33]. Moreover, besides identifying structural defects in teeth, OCT enables precise visualization and quantitative measurement of the periodontal pocket depth around the tooth and the assessment of peri-implant bone defects as a possible alternative to conventional probing [16,34]. Altogether, these versatile capabilities make OCT a promising tool for routine dental diagnosis, extending it to support comprehensive dental diagnosis and preventive measures, including caries detection.

OCT technology continues to advance toward clinical translation in dentistry. To facilitate chair-side diagnostics, hardware improvements have focused on miniaturization, portability, and enhanced intraoral accessibility—essential adaptations for a technology originally developed for ophthalmology [35]. Handheld probe-type OCT devices for imaging dental plaque and gingival tissues in vivo have been developed and clinically validated [36]. Furthermore, artificial intelligence integration is emerging to facilitate clinical interpretation of OCT images. Convolutional neural network-based and transformer-enhanced algorithms have been applied to automate assessment of alveolar bone levels and detection of demineralized lesions [37,38]. These technological advances provide a practical pathway toward routine clinical deployment of dental OCT.

This study has several limitations. First, this was an in vitro study using extracted teeth, which may not fully replicate the clinical environment in which factors such as saliva, patient movement, and varying levels of tooth surface contamination can affect diagnostic accuracy. Second, focusing on occlusal caries allowed for controlled conditions, but may limit the generalizability of the ability of OCT to detect caries in other types of lesions. Future studies should consider a broader range of carious lesions to comprehensively validate OCT’s diagnostic capabilities.

In conclusion, this in vitro study demonstrated that combining visual inspection with OCT enhances the diagnostic accuracy and sensitivity for detecting initial and moderate non-cavitated carious lesions compared to intraoral radiography. OCT’s ability to visualize subsurface demineralization and differentiate between infected and healthy tissues is advantageous for caries diagnosis, enabling more conservative and preventive treatment strategies. While challenges such as the in vitro study design and OCT learning curve remain, this study revealed the potential of OCT as a noninvasive, high-resolution imaging modality that demonstrates improved diagnostic accuracy for non-cavitated occlusal caries detection. Future clinical studies are required to validate these findings in vivo, assess their impact on treatment decisions and patient outcomes, and to facilitate the integration of OCT into routine dental diagnostics.

## Figures and Tables

**Table 1:** Criteria used for the discrimination between initial and moderate carious lesions.

	Initial Stage	Moderate Stage
Clinical visual examination	Changes in enamel are seen as a carious opacity or visible discoloration	Localized enamel breakdown or an underlying dentin shadow
ICDAS score	Code 1/2	Code 3/4
Lesion depth	Demineralization confined to enamel or less than the outer third of dentin	Demineralization penetrates more than the outer third of dentin, but still without surface cavitation
Recommended treatment	Non-operative care or periodic recall	Tooth-preserving operative care

**Table 2:** Diagnosis of non-cavitated lesions using two diagnostic approaches and the gold standard from the reference test.

	OCT-Based	Radiography-Based	Reference Test
Initial caries	56 (52.34%)	76 (71.03%)	64 (59.8%)
Moderate caries	51 (47.66%)	31 (28.97%)	43 (40.2%)

**Table 3:** Diagnostic performance of OCT- and radiography-based methods.

	OCT-Based	Radiographic-Based
Sensitivity (95% CI)	0.83 (0.70, 0.92) *	0.48 (0.35, 0.63) *
Specificity (95% CI)	0.76 (0.65, 0.85)	0.84 (0.74, 0.91)
Accuracy (95% CI)	0.79 (0.71, 0.86)	0.70 (0.61, 0.80)
AUC (95% CI)	0.80 (0.73, 0.88)	0.67 (0.58, 0.75)

* Indicates a statistically significant difference between the two diagnostic methods according to McNemar’s test (*p* < 0.05).

**Table 4:** The contingency table for the overall accuracy of OCT- and radiography-based methods.

	OCT Accurate	OCT Inaccurate
Radiograph Accurate	62	13
Radiograph Inaccurate	23	9

## Data Availability

The raw data supporting the conclusions of this article will be made available by the authors on request.

## Abbreviations

The following abbreviations are used in this manuscript:

ORCA-EFCD	European Organization for Caries Research and the European Federation of Conservative Dentistry
ICDAS	International Caries Detection and Assessment System
OCT	Optical Coherence Tomography
CCI	CariesCare International
SLD	Superluminescent diode
MEMS	Microelectromechanical system
CMOS	Complementary metal–oxide–semiconductor
AUC	Area under the curve
ROC	Receiver operating characteristic
